# Synthesis of water-soluble and bio-taggable CdSe@ZnS quantum dots

**DOI:** 10.1039/c7ra13400b

**Published:** 2018-02-23

**Authors:** G. Ramalingam, K. Venkata Saravanan, T. Kayal Vizhi, M. Rajkumar, Kathirvelu Baskar

**Affiliations:** Department of Nanoscience and Technology, Alagappa University Karaikudi Tamil Nadu India 630 003 ramanloyola@gmail.com +91 9445295572 +91 04565 225630; Department of Physics, Central University of Tamil Nadu Thiruvarur Tamil Nadu India 610 101 venketvs@cutn.ac.in; Department of Environmental Sciences, Bharathiyar University Coimbatore Tamil Nadu India 641 046; Entomology Research Institute, Loyola College Chennai-600 034 Tamil Nadu India suribaskar@hotmail.com

## Abstract

Many synthesized semiconductor QDs materials are formed using trioctylphosphine oxide (TOPO) but it requires high temperature, is very expensive and is also hydrophobic. Our study deals with selective syntheses of CdSe and core–shell CdSe/ZnS quantum dots (QDs) in aqueous solution by a simple heating and refluxing method. It is more hydrophilic, needs less temperature, is economically viable and is eco-friendly. Bio-ligands, such as thioacetamide, itaconic acid and glutathione, were used as stabilizers for the biosynthesis of QDs. A simplified aqueous route was used to improve the quality of the colloidal nanocrystals. As a result, highly monodisperse, photoluminescent and biocompatible nanoparticles were obtained. The synthesized QDs were characterized by XRD, FTIR, confocal microscopy, ultraviolet (UV) absorption and photoluminescence (PL). The size of synthesized QDs was observed as 5.74 nm and the core–shell shape was confirmed by using XRD and confocal microscopy respectively. The QD nanoparticles showed antibacterial activity against pathogenic bacteria. The QDs could be applied for biological labelling, fluorescence bio-sensing and bio-imaging *etc.*

## Abstract 2

Mystristic capped CdSe QDs with schematic diagram and formation mechanism of bio-taggable CdSe@ZnS QDs.

## Graphical abstract

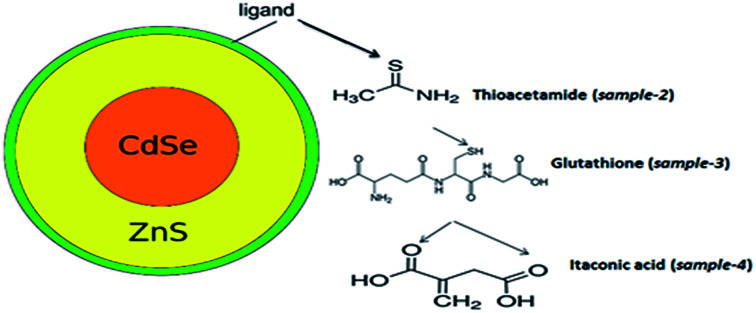

## 1. Introduction

Recently, semiconductor QDs were used for different applications ranging from optoelectronic to bio-molecular applications. Especially, colloidal quantum dots (QDs) are used in fluorescence, bio-sensing, bio-imaging and, most important, bio-diagnosis applications.^1,2^ For medical diagnosis purposes, usually organic dyes were used; these have less photostability and less sensitivity. To overcome these limitations, QDs open new prospects for long-term stability, multitargeting and high sensitivity for diagnosis as well as treatment. The QDs could be successfully used for ultrasensitive and multiple-target protein detection instead of organic dye fluorophores in different immune fluorescence assays, including the Enzyme-Linked Immunosorbent Assay (ELISA).^2^ Specifically, it has been shown that the sensitivity of a “sandwich” ELISA cancer testing technique can be improved by applying the spectral shift effect of a photoluminescence (PL) band in CdSe/ZnS core–shell QDs in conjugation with targets specific for cancer antigens.^3^

New trends, the study of colloidal semiconductor nanoparticles or quantum dots, have generated great interest because of their potential photonic applications from energy harvesting to biomedical applications.^2–5^ For all these applications, luminescent and water soluble QDs are required. It is well known that the photoluminescent emission intensity of CdSe quantum dots (QDs) increases several times when the CdSe cores are capped inside a shell with a higher band gap material.^6^ ZnS materials have a higher band gap of 3.54 eV. The combination of a core (CdSe) and a shell (ZnS) has good properties that increase the photoluminescence behaviour and other nanoparticle changes in the size and size distribution of the QDs can also subsequently change the luminescence and optical properties of the synthesized nanocrystals.^7^

Core/shell QDs have been widely investigated as fluorescent biomarkers, due to their photochemical stability and high brightness, which makes them a good alternative to organic fluorophores. It has been proved that over-coating the QDs with inorganic semiconductor materials can substantially increase the PL (quantum yield); chemical stability and photostability are by passivation of the surface non-radiative recombination sites.^8^ Core/shell QDs exhibit a lot of novel properties, making them attractive for experimental as well as practical applications.^9,10^ To improve the chemical stability and increase the PL QYs of CdSe QDs, core/shell or core/shell/shell QDs, such as CdSe/CdS, CdSe/ZnS and CdSe/CdTe, CdSe/CdS/ZnS and CdSe/ZnSe/ZnS, have been extensively studied.^11–16^ Furthermore, CdSe/ZnS core/shell QDs has become one of the best semiconductor QDs available for almost all biological applications.^17,18^ The CdSe/ZnS core/shell QDs increased QYs and improved chemical stability against oxidation compared to CdSe QDs.^8^ Consequently, core/shell type QDs, such as CdSe/ZnS, have been widely used in both optoelectronic and biological applications.^19–21^ The effect of spectral shift has been found to arise upon drying of the QDs dispersed in a buffer solution on a crystalline Si wafer. The shift in magnitude, which increases storage of the dried QD, sets atmospheric ambience for several days. Bio-conjugated QDs have increases much faster than the non-conjugated QDs, which enables us to distinguish the PL originating from the bio-conjugated QDs and that from the non-conjugated. This property can be used to improve the sensitivity of the QD luminescent tagging techniques.^8,18^

The most successful and well-developed method to prepare highly luminescent II–VI QDs is the ‘TOPO based hot-injection’ synthetic approach.^22–25^ However, in this method a high temperature is needed and the obtained QDs are insoluble in water, which limits their biological applications.^26^ Moreover, some of the key chemicals employed in organometallic synthesis are extremely toxic, pyrophoric, explosive and/or expensive, as reported by Peng and co-workers.^27–29^

Therefore, a number of surface functionalization studies have been developed to make QDs water soluble and biologically compatible.^27–32^ For biomedical applications, high-quality water-soluble quantum dots are required. QDs could be made directly in water, but often have narrow available size ranges and a wide size distribution (leads to a wide FWHM, full-width at half-maximum of the emission spectrum).^30–33,33,34,34–37^ In addition, the state-of-the-art of aqueous synthesis of QDs has been reviewed by Rogach *et al.*^35,38^ who illustrated the correlation between luminescence quantum efficiencies, luminescence life times and Stokes shifts of CdTe NC fractions. In general, the challenge is not only to make the high-quality hydrophobic quantum dots soluble in water, but also make them active in bio-conjugate reactions. Therefore, a number of surface functionalization studies need to be developed for water soluble and biologically compatible QDs. For biomedical applications, a high quality of water-soluble quantum dots are required.

In this study, a novel strategy is given for the selective synthesis of CdSe@ZnS quantum dots in aqueous solution by using three kinds of ligands, thioacetamide (TAA), itaconic acid (ITA) and glutathione (GSH) as stabilizer. This approach can be easily extended to the large-scale, aqueous-phase production of simple core and core–shell QDs. CdSe@ZnS QDs are biocompatible, monodispersed and stable under physiological conditions. The methods to prepare QDs using TAA, ITS, GSH are simple, eco-friendly and can be easily extended to large-scale, aqueous-phase production. These new bio-taggable QD systems are expected to find wide applications in biological detection and diagnostics. The QDs were studied for photophysics and physico-chemical properties *via* characterization using X-ray diffraction (XRD) analysis, FTIR spectroscopy techniques, laser confocal microscopy imaging, ultraviolet-visible absorption spectroscopy and photoluminescence behaviour. The anti-bacterial activity of the synthesized QDS was also assessed.

## 2. Experimental section

### 2.1 Synthesis techniques and tools used for characterization

The chemicals cadmium oxide (CdO), sodium selenide (Na_2_O_3_Se), hydrazine hydrate (N_2_H_4_·H_2_O), myristic acid (C_14_H_28_O_2_), zinc nitrate hexahydrate (Zn (NO_3_)_2_·6H_2_O), thioacetamide (C_2_H_5_NS), thiourea (CH_4_N_2_S), glutathione (C_10_H_17_N_3_O_6_S), itaconic acid (C_5_H_6_O_4_) and sodium hydroxide (NaOH) were purchased from Sigma-Aldrich with 99% purity. Ultra-pure water (Milli-Q, Millipore) was used throughout the experiments.

**Fig. 1:**
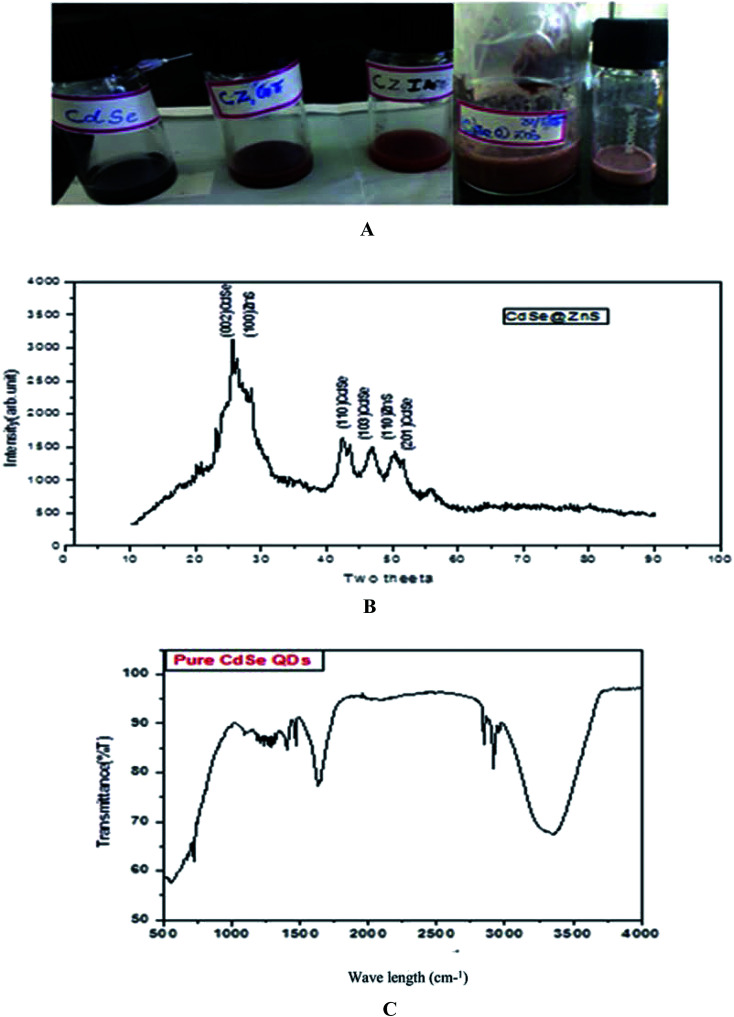
(A, top) as prepared quantum dot samples. (B, middle) XRD pattern of the CdSe@ZnS sample. (C, bottom) FTIR spectrum of Pure CdSe.

1 mmol of cadmium oxide (0.12 g) was dissolved in the capping ligands and 2.2 mmol of myristic acid (0.75 g). Cadmium oxide and myristic acid were mixed and heated up to 100 °C until a clear solution was obtained. Sodium selenide (Na_2_SeO_3_) was prepared by dissolving a solution containing 1 mmol Na_2_SeO_3_ (0.12 g) and 7 ml hydrazine hydrate for further dissolution. The transparent solution was a red colour. The solution was used as the selenium source for CdSe QDs. Finally, Se source was added to the mixture (Cd^2+^ : Se^2−^ – 1 : 1 ratio) and refluxed for 15 min at 180 °C. The red colour indicates CdSe QD formation. In a typical synthesis procedure of ZnS, 2.97 g of (Zn (NO_3_)·H_2_O) and 2.5 g of thioacetamide (TAA) were taken in the molar ratio 1 : 3 and each reagent was separately dissolved in 10 ml pf Millipore water and mixed thoroughly. The synthesis procedure of CdSe has already been described in our earlier report.^39^ The prepared ZnS and CdSe were mixed thoroughly with 25 ml of glutathione (GSH) and itaconic acid (ITA) for different samples. CdSe@ZnS QD core/shell QDs were produced by the controlled growth of ZnS over the CdSe QDs. The mixed solution was treated at 120 °C in a heating oven for a period of about 30 min and then allowed to cool down to room temperature naturally. Finally, a deep dark red powder was collected. After centrifugation and repeatedly washing with water and ethanol to remove residues present in the product, the sample was then dried at 120 °C for 3 h. The same procedure was followed for the entire sample with different organic capping ligands, such as TAA, ITA and GSH. The samples were labelled as CZTU, CZIA, and CZGT, respectively. Fig. 1(A) shows the prepared water soluble QDs at room temperature.

### 2.2 Characterization tools

A powder X-ray diffraction (PXRD) pattern was recorded on a rotation anode X-ray diffractometer (model Rich Seifer) with Ni-filtered CuK_α_ radiation (*λ* = 1.5406 Å). UV-vis absorption spectra and photoluminescence (PL) spectra were recorded by using a diode array spectrophotometer (Agilent technologies, serial no. CN22809757) and Cary Eclipse Fluorescence spectrophotometer (MY1507001), respectively. In the PL study, a 450 W xenon lamp was used as the excitation source and a photomultiplier tube with a resolution of 0.2 nm acted as the detector. The morphology of the water-soluble QDs was observed on a Confocal laser scanning microscope (CLSM-Leica TCS SP8) with a magnification of 60×. Images were captured in a DIC (differential interference contrast) mode and a fluorescence mode (488 nm lamp). The colour fluorescent images of the QDs were recorded on a digital colour camera (COOLPIX4300, Nikon, Japan) that was attached to an upright fluorescence microscope (Leica, DME, Germany). Antimicrobial activities of the synthesized quantum dots were performed against both Gram-positive (*Acinetobacter junii* and *Bacillus cereus*) and Gram-negative (*Flavobacterium columnare* and *Pseudomonas aeruginosa*) bacteria as described by the modified Kirby–Bauer disk diffusion method.^1^ Further, the QD compound induced peroxidation of lipids in human pathogens was determined by quantitative measurement of thiobarbituric acid (TBA)-reactive substances (TBARS).^58^

## 3. Results & discussion

### 3.1 Powder X-ray diffraction analysis

The powder XRD pattern of monodispersive CdSe/ZnS QDs is shown in Fig. 1(B). The evolution of the powder X-ray diffraction patterns during the growth of the shells around the spherical ZnS nanoparticles shows a hexagonal (wurtzite) phase of the CdSe. The high intensity (002) reflection of CdSe shows that the CdSe/ZnS QDs are identical with the *c*-axis of the wurtzite structure. All the observed peaks can be indexed to the wurtzite structure, with a lattice constant slightly compressed from that of bulk CdSe due to the CdSe/ZnS coating, which is very consistent with the characterized peaks of the wurtzite hexagonal CdSe/ZnS core–shell (JCPDS card no: 77-2307, 89-7385). A narrow peak (002) with a lower intensity of bare CdSe QDs was observed. The decrease in intensity of the (002) peak and the shift of all the peaks to higher angles are consistent with previous reports.^40^

The pattern of CdSe matches with that of the wurtzite structure and the peaks at 2*θ* = 25.7 (002), 42.4 (110), 47 (1 0 3) and 50.45 (201) are quite broad and overlapped due to the small crystal size. The straight lines show the position of diffraction peaks for CdSe powder having a pure hexagonal structure. ZnS nanocrystals can possibly crystallize in cubic as well as hexagonal structures, where the hexagonal structure is favourable at high temperatures. For CdSe/ZnS the diffraction peaks are more emergent, but not with much change in the FWHM of the respective peaks. The broad hump in the region 42 to 50 is clearly visible and is interpreted as the superposition. The peaks angle at 25, 42.4, 47, 50.45 and the corresponding diffraction plane (1 1 0) for the entire three peak angle is the same, which confirms the overlapping of the CdSe/ZnS structure. Further, the XRD peaks are broadened due to their small size distribution. From the Debye–Scherrer formula (Table 1), the crystalline size was determined from all the major and minor peaks. The average particle size was found to be 5.754 nm. However, high resolution TEM results will confirm the particle size.

**Table 1:** Particle Size Determination

Plane of index	2*θ*	FWHM (*β*)	*D* = 0.9*λ*/*β* cos *θ* (nm)
(002) CdSe, (100) ZnS	25.7°	6.4485	1.316
(110) CdSe	42.4°	13.0521	7.0
(103) CdSe, (110) ZnS	47.0°	0.9438	9.6
(110) ZnS, (201) CdSe	50.45°	1.7973	5.1
Average crystalline size (*D*) = 5.754 nm			

### 3.2 FTIR & confocal microscopy

#### 3.2.1 FTIR and confocal microscopic study of pure CdSe

Fig. 1(C) shows the FT-IR spectrum of the as-prepared CdSe NPs. Initially, the sample was washed with absolute ethanol and hot distilled water several times and then dried in a vacuum for FTIR analysis. Fig. 1(C) illustrates the presence of a broad peak at 3356 cm^−1^, which is assigned to OH stretching of intramolecular hydrogen bonds due the presence of a meagre amount of H_2_O in the sample. The N–H stretching vibration peak observed at 3282 cm^−1^ is due to the presence of hydrazine hydrate in the sample.

The peak observed at 1642 cm^−1^ is assigned to the OH of water absorbed from the molecular precursors. The C–N stretching vibration peak positioned at 1093 cm^−1^, is due to the interaction of myristic acid with the hydrazine hydrate and the regular periodic structure of the molecular precursors. The exact mechanism for the formation of CdSe nanoparticles is still unclear, but it can be reasonably concluded that an appropriate ratio of solvent volume might play a significant role in the formation of CdSe NPs. On the basis of the above observations, a growth mechanism of the CdSe NPs is proposed. In the present work, the Se source can be easily converted into Se^2−^ by N_2_H_4_, which will result in a high monomer concentration. In the initial step, hydrazine hydrate (N_2_H_4_·H_2_O) complexes with Cd^2+^ and forms a transparent soluble complex solution, which effectively decreases the concentration of Cd^2+^, avoiding the precipitation of CdSeO_3_, and thereby providing a more homogeneous solution environment for the reaction.

The chemical reaction involved in the entire synthesis of CdSe NPs could be formulated as the following:2Se + N_2_H_4_ + 4OH → 2Se^2−^ + N_2_ + 4H_2_O2Cd^2+^ + 4OH^−^ → 2CdO + 2H_2_OCdO + Se^2−^ + H_2_O → CdSe + 2OH^−^2Cd^2+^ + 6OH^−^ → 2Cd(OH)_3_^−^Cd(OH)_3_^−^ + Se^2−^ → CdSe + 3OH^−^

**Fig. 2:**
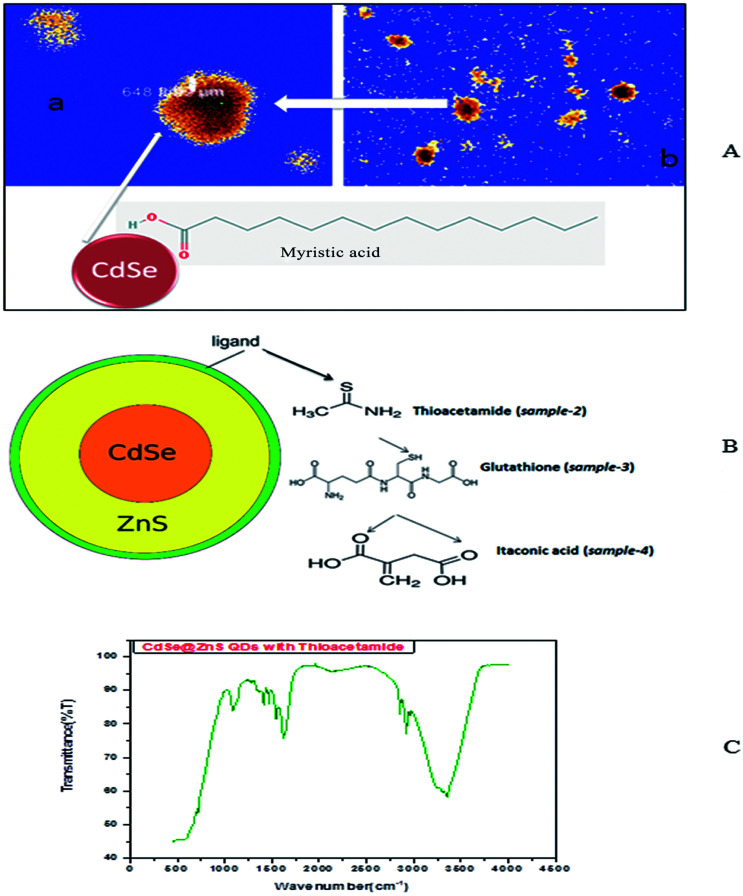
(A) Confocal images of myristic capped CdSe QDs. (B) Formation mechanism of bio-taggable CdSe@ZnS QDs. (C) FTIR spectrum of thioacetamide taggable CdSe@ZnS QDs.

So, the application of N_2_H_4_ as the coordination agent is determinable for this phase of the products. Thus, it can be concluded that the complexing ability of groups containing atom N (such as NH_2_ or NH_3_) can effectively determine the final phase of the products. Compared to the CdO deposit, it is easier for Cd(OH)_3_^−^ to release Cd^2+^, which can facilitate the growth of nanoparticles under non-equilibrium kinetic growth conditions with a high monomer concentration. A similar phenomenon was found during the preparation of PbSe and Cu_2_Te nanostructures using N_2_H_4_·H_2_O as the complexing agent and the exact mechanism was fully understood.^41,42^ Confocal microscopy allows the direct imaging of nanoparticles, which provides authentic information on the distribution, size and morphology of the nanocrystallites. The low-magnification confocal images of CdSe, as shown in Fig. 2(A and B), confirms the uniform size and shape distribution of CdSe QDs. The influence of hydrazine hydrate controls the uniform distribution of CdSe QDs. The reverse micelle-assisted wet chemical method, hydrazine hydrate and myristic acid was used as both reducing and templating agent, and its presence was found to favour the formation of spherical doted-like structure. Myristic acid plays a significant role in the formation of distinct, monodisperse, spherical uniform QDs. The schematic and confocal image is shown in Fig. 2(A). Myristic acid helps to prevent agglomeration of free QDs, which is clearly evident from the confocal images. The general synthesis model and formation mechanism of the as prepared other three samples are shown in Fig. 2(B).

#### 3.2.2. FTIR and confocal microscopic studies of thioacetamide capped CdSe@ZnS

The FTIR spectra of the synthesized CdSe/ZnS nanoparticles are shown in the Fig. 2(C). The strong peak at 3348 cm^−1^ for CdSe indicates hydroxyl bond (O–H) stretching, which confirms that the OH groups remain intact on the surface. The weak IR peaks at 2919 cm^−1^ for CdSe and 2141 cm^−1^ for CdSe/ZnS correspond to the CH_2_–S stretching. These peaks support the onset of covalent bonds between S and Zn^2+^ ions at the nanoparticle surface, thus binding the ZnS shell onto the CdSe core. The narrow and strong peaks at 1622 cm^−1^ and 1396 cm^−1^ for CdSe and short peaks at 1541 cm^−1^ and 1470 cm^−1^ for CdSe/ZnS nanoparticles correspond to the COO vibrations. The bands positioned at 1079 cm^−1^ correspond to the C–O stretching. Further, from the FT-IR spectrum it was found that the thioacetamide ligand was coordinated to the Cd^2+^ ion forming a Cd–thioacetamide nanocomposite. The N–H stretching vibrations shifted from 3348 and 2919 cm^−1^ of thioacetamide to 3348 cm^−1^ of the Cd–thioacetamide nanocomposite, while the peak at 2919 cm^−1^ corresponding to the N–H bond disappeared. The C–N stretching vibrations shifted from 1622 and 1470 cm^−1^ due to presence of Cd-thioacetamide nanocomposite. The change in bonding energy of N–H reveals the coordination of cadmium ions and thioacetamide molecules. It can be concluded that nitrogen atoms of the thioacetamide molecules were involved in the Cd–thioacetamide nanocomposite formation by donating a lone pair of electrons from the nitrogen atoms, to form a coordination compound with the vacant d-orbital of Cd cations.

**Fig. 3:**
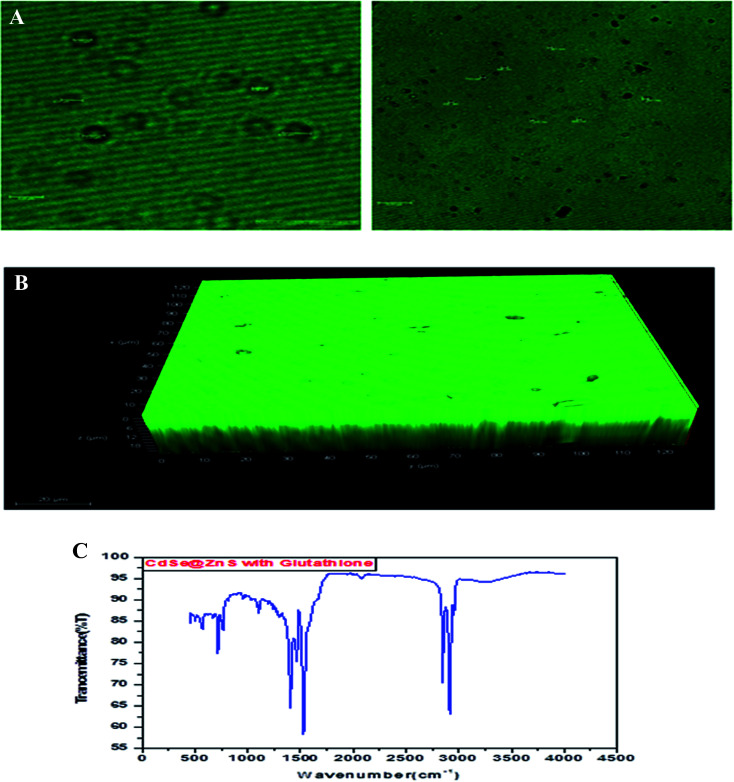
(A) Confocal images of thioacetamide capped CdSe@ZnS QDs. (B) 3D confocal images of thioacetamide capped CdSe@ZnS QDs. (C) FT-IR spectrum of glutathione taggable CdSe@ZnS QDs.

The highly water soluble and biocompatible thioacetamide capped CdSe/ZnS QDs of a narrow size distribution were synthesized without using any additional stabilizer. The confocal images of CdSe@ZnS QDs are shown in Fig. 3(A and B). This pattern clearly indicates that the core shell like quantum nanoparticles with uniform monodisperse, agglomeration free QDs were formed. Fig. 3(B) shows the 3D image. We have chosen thioacetamide amino acid to cap CdSe and ZnS nanoparticles as it passivates on the surface states more effectively than other thiols. Further, thioacetamide provides biologically active end groups for possibly targeting specific cell sites. Variable PL maxima with respect to particle size can lead to the development of suitable fluorescent biological probes. Therefore, efforts have been taken to establish the feasibility to prepare narrow size CdSe/ZnS QDs. Thioacetamide plays three essential roles in the present study; it acts as a source for sulphide ions, as a growth moderator and as a stabilizer.

#### 3.2.3 FTIR and confocal microscopic studies of glutathione-capped-CdSe@ZnS QDs

To verify the existence of GSH on the surface of the as-prepared QDs as a capping agent and stabilizer, an FTIR analysis was carried out. Fig. 3(C) illustrates that the FTIR absorption bands of free GSH at 3315 and 2111 cm^−1^ are ascribed to N–H stretching bands (NH^3+^), whereas the peaks at 2111 and 1635 cm^−1^ are assigned to S–H and –NHR groups, respectively. By contrast, the disappearance of the S–H stretching vibrational peak, the near disappearance of the N–H stretching bands, the clear weakening of the amide bond, and the peaks at 1635 and 1118 cm^−1^ were assigned to C_1/4_O, C–O and C–N groups, respectively, which confirms the presence of –COOH and –NH_2_, indicating that GSH combines onto the surface of the QDs through the S–H and –NHR groups.

**Fig. 4:**
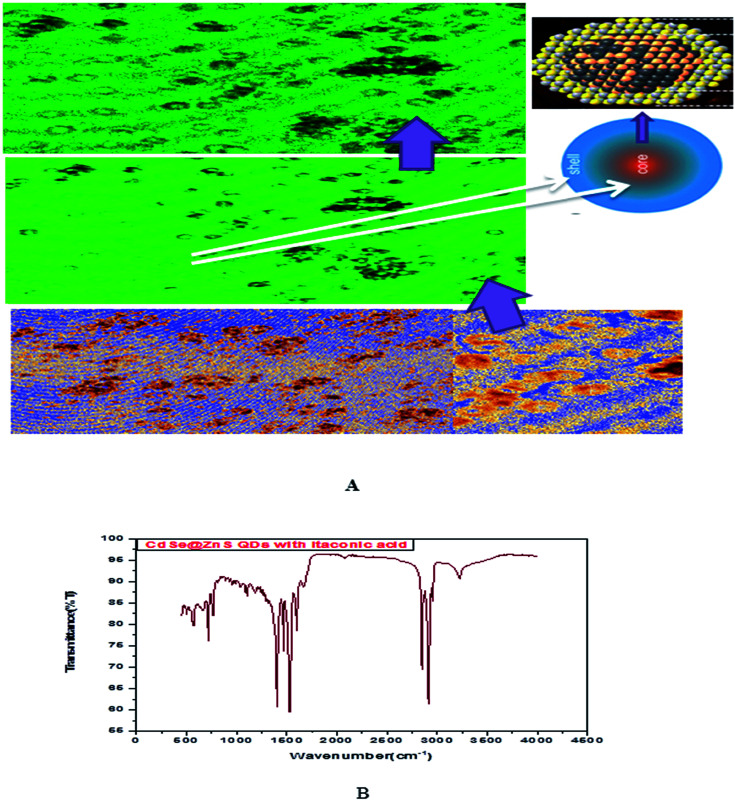
(A) Confocal images and formation mechanism of GSH capped CdSe@ZnS. (B) FT-IR spectrum of itaconic taggable CdSe@ZnS.

Glutathione-capped-CdSe@ZnS QDs have good water solubility, high quantum dots (QY) and promising compatibility with biological application. Moreover, these sensors, based on the quenching of QDs PL emission, also respond to other metal ions to varying degrees. Consequently, QD-based sensor pre-treatments with different certain specific ligands (*e.g.*, mercaptosuccinic acid, 2,2-dithiodibenzoic acid) are often required for the purpose of a remarkable responsiveness to a certain metal ion. GSH contains an amino group, carboxyl group, mercapto group, amide group and other electronic ligand groups, which might play a significant role in the detoxification process when binding to the toxic intracellular metal ions. A large number of studies demonstrate that toxic metal ions (such as Hg, Cu, Cd, Ni) could dramatically reduce the content of intracellular GSH.^43,44^Fig. 4(A) gives clear information for understanding the core shell like CdSe@ZnS QDs. The facile synthesis of glutathione-capped CdSe@ZnS QDs is simple and cost-effective compared to the conventional organometallic approaches. It represents the first direct synthesis of blue fluorescent QDs in aqueous solution. The approach can be easily scaled up for the commercial production of alloyed nanocrystals of various compositions. The glutathione molecule acts as an excellent reagent, stabilizer and sulphur source for the synthesis of high luminescence CdSe and core–shell CdSe/ZnS QDs in aqueous solution.

Using GSH as the stabilizer and sulphur source for QD synthesis makes the synthesized CdSe@ZnS QDs eco-friendly and biocompatible when used as biological probes, since GSH is found in the cell cytosol and mother aqueous phases of the living system. Similarly, Ying-Fan Liu *et al.*^45^ developed a selective synthesis of CdTe and high luminescence CdTe/CdS quantum dots. These methods illustrate that high-quality CdTe and CdTe/CdS QDs prepared with GSH in the aqueous phase is extremely simple, convenient and highly efficient because GSH easily decomposes to release S at a low-temperature (100 °C) refluxing for 10 min to several hours. The aggregation-induced emission (AIE) was found using a thiolate complex proved by many researchers^46–48^ and contributes a significant study about the formation of a core–shell using a thiolate complex like GSH. The aggregation of metal–thiolate complexes promoted intra and intercomplex aurophilic interaction between the closed core–shell quantum dots model. Further, the aurophilic bonds in turn provided the impetus for the aggregation of denser and more rigid aggregate nanoparticles. The metal/cation aggregation method exploited the high affinity of electrostatic and coordination interaction between certain multivalent cations (*e.g.*, Cd) and monovalent carboxylic anions (from GSH) in the complexes to form inter and/or intracomplex cross-links, like CdSe–GSH–ZnS formation. Besides neutralizing the negative charge on the complexes, the cross-link also brought the Au(i)–thiolate complexes closer and facilitated the formation of aurophilic bonds and dense aggregates.^48,49^

#### 3.2.4 FTIR and confocal microscope studies of itaconic acid-capped-CdSe@ZnS QDs

FTIR analysis of itaconic acid capped QD nanoparticles was conducted and results are shown in Fig. 4(B). The amino group peak is at 1527 (I) and the amide (I) bond peak at 1405 cm^−1^ (II). These amino groups may be used to further attach biomolecules to the itaconic acid QD nanoparticles. The synthesized QDs are observed to remain stable up to pH 9 with no visible sign of agglomeration.

**Fig. 5:**
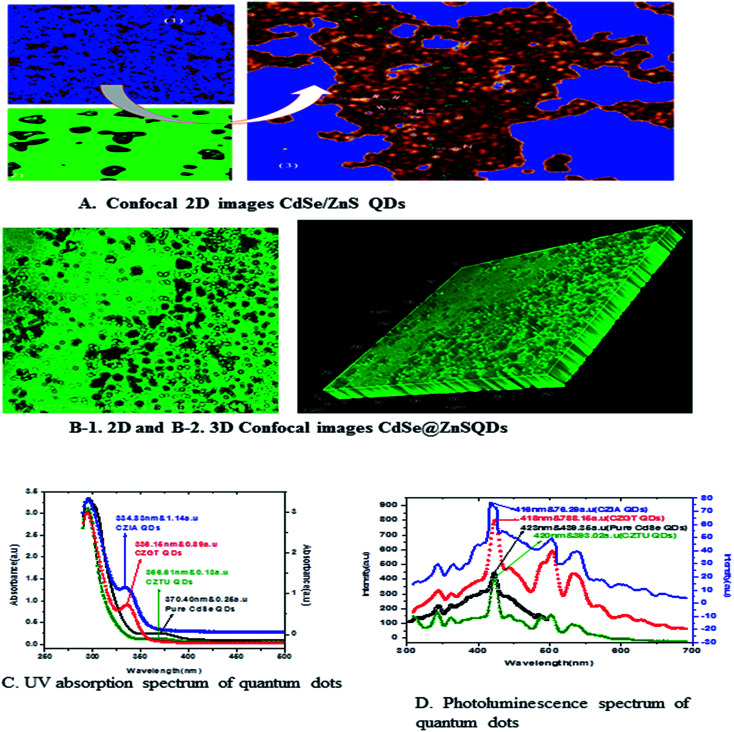
(A) Confocal 2D images of CdSe/ZnS QDs. (B-1) 2D and (B-2) confocal 3D image of itaconic taggable CdSe@ZnS QDs. (C&D) UV & PL spectra of CdSe@ZnS QDs.

Fig. 5 shows the formation of core shell CdSe@ZnS QDs. The prepared nanocrystals had distinct core–shell structure, separated and purified nanocrystals before and after the growing of the product. To achieve good reproducibility in the synthesis of core–shell QDs, the processing stage was optimized specially. The growth of the ZnS shell on the CdSe core is accompanied by a significant broadening of particle size distribution. We found that the parameter (ITA) affects the size distribution of colloidal nanoparticles. ITA is used as a function monomer and stabilizer in a true system CdSe/ZnS core/shell structure during synthesis. The 2D and 3D images of itaconic acid capped QDs are shown in Fig. 5(B-1 and B-2).

### 3.3 Optical absorption studies

Fig. 5(C) shows the absorption spectra of the as prepared CdSe and CdSe/ZnS QDs with different bio-ligands, such as ITA, GSH and TAA. For pure CdSe QDs the absorption peak is centred around 370 nm, which shows a lower absorption intensity scale as indicated on the right side. After coating of the ZnS shell, a significant absorption was observed under short wavelengths of 334, 336 and 366 nm for CZIA, CZGT and CZTU QDs, respectively. The sharp excitonic absorption peak indicated a narrow size distribution of the product. The results indicated that the high intensity would be good for forming a narrow size distribution of the product.^25^ The absorption spectra indicated a blue shift for the core-shells as compared to the core CdSe (pure material).

### 3.4 Photoluminescence spectral analysis

Generally, the PL intensity of bare water-soluble CdSe NPs is weak, but after coating with shells, nanoparticles have relatively favourable optical properties because of the reducing non-radioactive recombination, by confining the wave function of electron–hole pairs to the interior of the NPs. Therefore, CdS, ZnS and ZnSe with a larger band gap have been used as an inorganic shell material to coat the CdSe core NPs.^50^

The photoluminescence spectra of Fig. 5(D) show the pure CdSe and CdSe/ZnS with bio-ligand tagged QDs. For pure CdSe QDs, the emission peak centres around 423 nm, which shows less emission intensity. After coating the ZnS shell, a significant photo brightening was observed under short wave lengths of 416, 418 and 420 nm for CZIA, CZGT and CZTU QDs, respectively. The brightness of the CdSe/ZnS QD core–shell was observed to be enhanced when compared to that of the pure CdSe nanocrystals. The brightening process is accompanied with a slight blue-shift of the PL spectra (Fig. 5(D)), which is similar to the photo-brightening previously observed in nanocrystals.^51–53^ This implies that the photo-brightening process is likely due to the photo-oxidation of the surface ZnS shell layer, as documented previously.^13^ The absorption and emission edge is a clear indication of the visible light response of the particles indicating that the material can be used for various fluorescence applications. In addition, the material can be used as promising biological labels and other biological applications, due to its lower toxicity and biocompatibility. Better protection of the surrounding medium from the toxic element present in the emitting CdSe core is provided by the non-toxic ZnS shell which makes the material biocompatible. The photoluminescence quantum yield increased with increasing shell thickness, which can be confirmed by XPS and TEM studies.

The photoluminescence has multiple peaks because the sample solution has a low concentration at a high temperature synthesis or *vice versa* and changes in particle size. It has been reported that the core shell quantum dots overcoated with higher band gap inorganic materials exhibits a higher PL quantum yield compared to the uncoated QDs, perhaps by the elimination of surface non radiative recombination defects.^54,55^

#### 3.4.1 Antibacterial activity

**Fig. 6:**
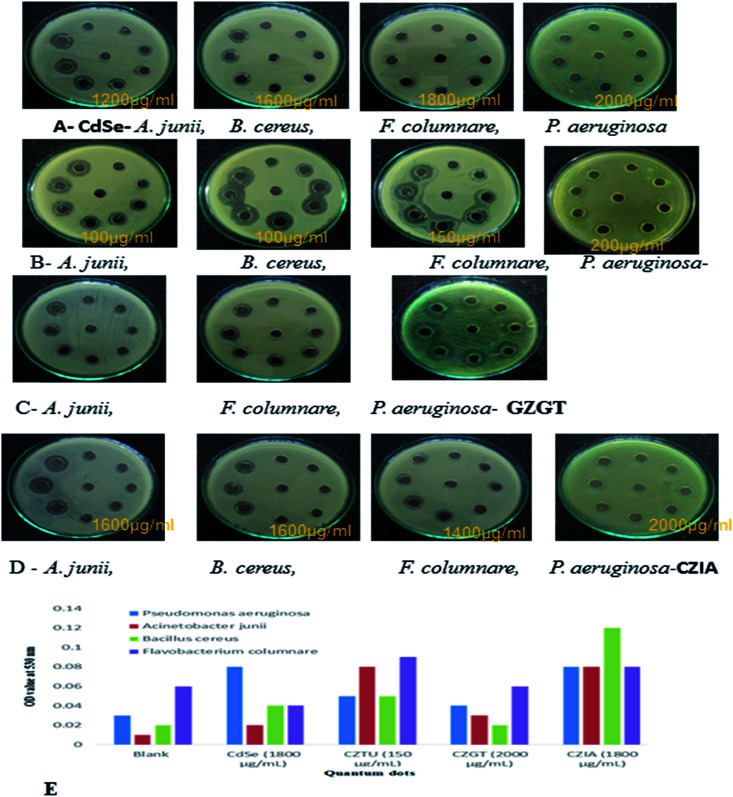
(A, B, C & D) antibacterial testing CdSe@ZnS QDs. (E) TBARS production in QD treated bacterial pathogens.

The antibacterial activity of quantum dots was evaluated against human pathogens (two Gram-positive and negative) including *A. junii*, *B. cereus*, *F. columnare* and *P. aeruginosa* using MH agar plate (Table 2 and Fig. 6(A–D)). Among the four quantum dots tested, CZTU exhibited better antimicrobial activities against the tested pathogens. At concentrations ranging from 100 to 200 μg ml^−1^, CZTU showed a 10 to 13 mm zone inhibition against all bacterial strains. Further, this zone became larger as the CZTU concentration increased for almost all of the pathogenic bacteria tested (data not shown). Other quantum dots showed very weak antibacterial activity at concentrations ranging from 100 to 1000 μg ml^−1^, but at higher concentrations ranging from 1400 to 2000 μg ml^−1^ they showed moderate antibacterial activity. For instance, at concentrations ranging from 1400 to 2000 μg ml^−1^, CdSe and CZIA showed zones of inhibition of 9 to 12 mm and 11 to 14 mm, respectively, against the bacterial strains tested. This result clearly shows that CZTU is a better compound than the other quantum dots.

Antimicrobial effects are usually accompanied by a change in the bacterial morphological, physiological and biochemical features. Though various mechanistic approaches were proposed to explain the anti-microbial activity of metal containing nanomaterials including the release of metal ions and cellular membrane dysfunction, *etc.*, the generation of reactive oxygen species (ROS) has been reported as the major causative factor because the accumulation of ROS causes damage to DNA, proteins and lipids, which leads to disorganization, dysfunction and damage of the membranes and proteins.^56,57^ Particularly, the oxidation of lipids due to ROS impairs membrane function, decreases fluidity, increases permeability to ions and potentially ruptures the membrane.^50^ Hence, in the present study, the oxidation of lipids by ROS was investigated in quantum dot treated Gram-positive (*Acinetobacter junii* and *Bacillus cereus*) and Gram-negative (*Flavobacterium columnare* and *Pseudomonas aeruginosa*) bacteria after 24 h from the start of the bacterial growth (Fig. 6(E)). Compared to the control group (*i.e.*, without quantum dots in culture media), the thiobarbituric acid reactive substance (TBARS) for the quantum dot treated group was observed to be of a higher activity, which indicates an increase in the ROS generation due to the presence of quantum dots in the culture media. However, it is interesting to note that even at a concentration of 150 μg ml^−1^, CZTU generated a significant amount of TBARS in the selected pathogens. On the other hand, the TBARS level measured for the tested pathogens treated with CZGT (2000 μg ml^−1^) was found to be lower. The measured TBARS equivalent, which was equivalent to the generation of ROS, is in good agreement with the value given in Table 2 concerning antibacterial activity of various quantum dots. Our results are in agreement with recent reports on the enhanced toxicity of pure CdSe, CZTU, CZGT and CZIA samples to bacterial strains.^58^ Findings from this study clearly indicate that CZTU has the potential to be developed as a novel antimicrobial agent. However, further studies including the analysis of the mechanism of interaction of QDs with the microbial cells are required in order to explore in-depth molecular mechanisms through which QDs control pathogens. Since the higher concentrations of metal containing nanomaterials, for example, silver nanoparticles, are highly toxic and can cause various health and environmental problems, studies on the long-term toxic effects of QDs and its biocompatibility using animal models and clinical studies are also essential before QDs are brought into the healthcare field.^59^

## 4. Conclusions

We have developed a novel synthesis of luminescent semiconductor nanocrystals consisting of a CdSe core and ZnS as the outer shell. The sizes of the QDs were successfully controlled by an environmental friendly solvent. The powder XRD and EDX patterns confirm the hexagonal crystalline structure, purity and composition of the obtained product. The synthetic QD nanocrystals are significantly simple and effectively water soluble. Amine groups are significantly weaker bonding sites; however, the bio-ligand QDs have a bidentate chelating moiety, which showed a high affinity for metal atoms and increased stability of the nanocrystal. The nanocrystals exhibited significantly moderate luminescence and the same optical spectra as the CdSe/ZnS core–shell nanocrystals. In addition, the hydroxyl group is considered as a common type of biocompatible functional group, which has a low nonspecific binding for biomolecules. Furthermore, the chemistry related to CdSe/ZnS QD nanocrystals can be used for several applications, such as biological labelling, fluorescence bio-sensing, bio-imaging, *etc.*

## Conflicts of interest

We declare that we have no conflict of interest.

## Supplementary Material
